# HIV Services and Outcomes During the COVID-19 Pandemic — United States, 2019–2021

**DOI:** 10.15585/mmwr.mm7148a1

**Published:** 2022-12-02

**Authors:** Karen W. Hoover, Weiming Zhu, Zanetta C. Gant, Kevin P. Delaney, Jeffrey Wiener, Neal Carnes, Dominique Thomas, John Weiser, Ya-Lin A. Huang, Laura W. Cheever, Athena P. Kourtis

**Affiliations:** ^1^Division of HIV Prevention, National Center for HIV, Viral Hepatitis, STD, and TB Prevention, CDC; ^2^HIV/AIDS Bureau, Health Resources and Services Administration, Rockville, Maryland.

Increasing HIV testing, preexposure prophylaxis (PrEP), and antiretroviral therapy (ART) are pillars of the federal Ending the HIV Epidemic in the U.S. (EHE) initiative, with a goal of decreasing new HIV infections by 90% by 2030.* In response to the COVID-19 pandemic, a national emergency was declared in the United States on March 13, 2020, resulting in the closure of nonessential businesses and most nonemergency health care venues; stay-at-home orders also limited movement within communities (*1*). As unemployment increased during the pandemic (*2*), many persons lost employer-sponsored health insurance (*3*). HIV testing and PrEP prescriptions declined early in the COVID-19 pandemic (*4*–*6*); however, the full impact of the pandemic on use of HIV prevention and care services and HIV outcomes is not known. To assess changes in these measures during 2019–2021, quarterly data from two large U.S. commercial laboratories, the IQVIA Real World Data — Longitudinal Prescription Database (IQVIA),^†^ and the National HIV Surveillance System (NHSS)^§^ were analyzed. During quarter 1 (Q1)^¶^ 2020, a total of 2,471,614 HIV tests were performed, 190,955 persons were prescribed PrEP, and 8,438 persons received a diagnosis of HIV infection. Decreases were observed during quarter 2 (Q2), with 1,682,578 HIV tests performed (32% decrease), 179,280 persons prescribed PrEP (6% decrease), and 6,228 persons receiving an HIV diagnosis (26% decrease). Partial rebounds were observed during quarter 3 (Q3), with 2,325,554 HIV tests performed, 184,320 persons prescribed PrEP, and 7,905 persons receiving an HIV diagnosis. The proportion of persons linked to HIV care, the number who were prescribed ART, and proportion with a suppressed viral load test (<200 copies of HIV RNA per mL) among those tested were stable during the study period. During public health emergencies, delivery of HIV services outside of traditional clinical settings or that use nonclinical delivery models are needed to facilitate access to HIV testing, ART, and PrEP, as well as to support adherence to ART and PrEP medications.

Data from four data sources were used to estimate HIV service use and outcomes by quarter: 1) LabCorp, 2) Quest Diagnostics, 3) IQVIA, and 4) NHSS. Combined LabCorp and Quest Diagnostics laboratory data were analyzed to estimate the number of HIV tests performed during 2019–2021; Current Procedural Terminology codes were used to identify HIV antigen and antibody test results and HIV RNA test results. Laboratory data were also used to estimate the number of HIV viral load tests performed and the proportion of those tests indicating viral load suppression. IQVIA data on antiretroviral drugs dispensed by U.S. retail pharmacies and mail-order pharmacies during 2019–2021 were analyzed using a validated algorithm to estimate the number of persons prescribed PrEP or ART (*7*). Laboratory and IQVIA data were analyzed to assess the change from each quarter to the following quarter in the number of HIV tests and persons prescribed PrEP during 2019–2021, stratified by age group (15–24, 25–34, and ≥35 years). NHSS data from 2019–2020 were analyzed to identify the number of persons who received a diagnosis of HIV infection and the proportion of those persons linked to care within 1 month of diagnosis** as well as to assess the quarterly change in the number of persons who received an HIV diagnosis during 2019–2020, by age group, race and ethnicity, and transmission category. Incomplete race and ethnicity data and no transmission data were available in either the laboratory or IQVIA data; in addition, the number of persons who received an HIV diagnosis and the percentage linked to care were not available for 2021. Poisson regression models were used to assess trends in service use and outcomes by calculating the estimated quarterly percent change (EQPC) during 2019–2021 and 95% CIs; these models were also used to assess whether changes in the number of HIV tests and number of persons prescribed PrEP from Q1 to Q2 during 2020 differed significantly among age groups. This activity was reviewed by CDC and was conducted consistent with applicable federal law and CDC policy.^††^

The number of HIV tests and number of persons prescribed PrEP decreased early in the COVID-19 pandemic but started to rebound by mid-2020. During 2020, the number of HIV tests decreased 32% from Q1 (2,471,614) to Q2 (1,682,578) but increased in Q3 to 2,325,554 (2019–2021 EQPC = 0.33%) (Table 1). Similarly, during 2020, the number of persons prescribed PrEP decreased 6% from Q1 (190,955) to Q2 (179,280) but increased to 184,320 in Q3 (2019–2021 EQPC = 3.45%). Following a similar pattern, during 2020, HIV diagnoses decreased 6% from Q1 (8,438) to Q2 (6,228) but increased to 7,905 in Q3 (2019–2020 EQPC = −3.99%). The proportion of persons linked to HIV care, the number who were prescribed ART, and the proportion with a suppressed viral load test result among those tested was stable during the study period. Among persons who received a diagnosis of HIV infection, the percentage who were linked to care did not vary during 2019–2020, ranging from 88.0% to 89.4% (2019–2020 EQPC = 0.24%). During 2020, viral load tests performed decreased 20% from Q1 (259,026) to Q2 (206,586) but increased to 252,643 in Q3 (2019–2021 EQPC = 0.45%). The number of persons prescribed ART did not vary (2019–2021 EQPC = 0.24%). Similarly, the proportion of tests indicating viral load suppression did not vary and ranged from 86.7% to 89.0% (2019–2021 EQPC = 0.26%).

**TABLE 1:** HIV testing, preexposure prophylaxis, HIV diagnoses, linkage to HIV care, antiretroviral therapy, and viral suppression, by quarter* — United States, 2019–2021

HIV service or outcome	No. or % (% change from previous quarter)												2019–2021 EQPC, % (95% CI)
	2019				2020				2021				
	Q1	Q2	Q3	Q4	Q1	Q2	Q3	Q4	Q1	Q2	Q3	Q4	
No. of HIV tests^†,§^	2,101,633 (—)	2,523,317 (20.1)	2,572,963 (2.0)	2,451,303 (−4.7)	2,471,614 (0.8)	1,682,578 (−31.9)	2,325,554 (38.2)	2,274,593 (−2.2)	2,346,191 (3.1)	2,646,562 (12.8)	2,643,539 (−0.1)	2,453,114 (−7.2)	0.33 (0.31 to 0.34)
No. of persons prescribed PrEP^¶^	159,434 (—)	168,543 (5.7)	176,180 (4.5)	181,016 (2.7)	190,955 (5.5)	179,280 (−6.1)	184,320 (2.8)	187,478 (1.7)	193,587 (3.3)	215,715 (11.4)	236,323 (9.6)	243,515 (3.0)	3.45 (3.41 to 3.49)
No. of persons with diagnosed HIV infection**^,††^	9,488 (—)	9,431 (−0.6)	9,164 (−2.8)	8,392 (−8.4)	8,438 (0.5)	6,228 (−26.2)	7,905 (26.9)	7,758 (−1.9)	NA	NA	NA	NA	−3.99 (−4.31 to −3.67)^§§^
% of persons linked to care**^,††,¶¶^	88.0 (—)	87.9 (−0.1)	88.4 (0.6)	88.5 (0.1)	87.8 (−0.8)	89.2 (1.6)	89.4 (0.2)	89.3 (−0.1)	NA	NA	NA	NA	0.24 (−0.12 to 0.60)^§§^
No. of viral load tests^†^	225,149 (—)	270,189 (20.0)	269,265 (−0.3)	261,143 (−3.0)	259,026 (−0.8)	206,586 (−20.2)	252,643 (22.3)	250,823 (−0.7)	259,659 (3.5)	273,282 (5.2)	265,562 (−2.8)	254,675 (−4.1)	0.45 (0.42 to 0.48)
% with suppressed viral load^†,^***	86.7 (—)	87.2 (0.6)	87.3 (0.1)	87.8 (0.6)	87.3 (−0.6)	88.9 (1.8)	88.9 (0)	88.9 (0)	89.0 (0.1)	88.9 (−0.1)	88.8 (−0.1)	89.4 (0.7)	0.26 (0.23 to 0.30)
No. of persons prescribed ART^¶^	586,169 (—)	591,874 (1.0)	600,396 (1.4)	603,634 (0.5)	615,339 (1.9)	613,100 (−0.4)	600,336 (−2.1)	596,251 (−0.7)	604,627 (1.4)	605,727 (0.2)	609,394 (0.6)	611,884 (0.4)	0.24 (0.22 to 0.26)

**Abbreviations:** ART = antiretroviral therapy; EQPC = estimated quarter percentage change; PrEP = preexposure prophylaxis; Q1 = quarter 1; Q2 = quarter 2; Q3 = quarter 3; Q4 = quarter 4.

* Quarters were defined as Q1 (January 1–March 31), Q2 (April 1–June 30), Q3 (July 1–September 30), and Q4 (October 1–December 31).

^†^ Commercial laboratory testing data from LabCorp and Quest Diagnostics, 2019–2021.

^§^ HIV antigen/antibody testing data were missing for January 2019 from LabCorp; therefore, the total number of HIV tests in Q1 2019 is underreported. The EQPC for HIV testing was calculated for Q2 2019 through Q4 2019.

^¶^ IQVIA Real-World Data — Longitudinal Prescription Database, 2019–2021.

** National HIV Surveillance System, 2019–2020.

^††^ Data included in this study are from 46 jurisdictions (45 states and the District of Columbia) that had complete laboratory reporting for all data years. Linkage to care was defined as having one or more CD4 or viral load tests within 1 month of the HIV diagnosis. Data include 53 and 17 cases with missing month of HIV diagnosis for 2019 and 2020, respectively.

^§§^ EQPC calculated for 2019–2020. The number of persons who received an HIV diagnosis and the percentage linked to care were not available for 2021.

^¶¶^ Data include 48 and 17 cases with missing month of HIV diagnosis for 2019 and 2020, respectively.

*** Suppressed viral load calculation is for persons who had a viral load test result.

During 2020, among all age groups, persons aged ≥35 years experienced the largest quarter-to-quarter decrease in number of HIV tests from Q1 (1,076,548) to Q2 (660,593), representing a 39% decline (Figure). During the same period, persons aged 15–24 years experienced the largest quarter-to-quarter decrease in receipt of PrEP prescriptions (from 17,909 to 16,316, a 9% decrease). Among all racial and ethnic groups and HIV transmission categories, the number of persons who received a diagnosis of HIV infection in 2020 decreased from Q1 to Q2 (range = −21.1 [White] to −29.4 [Other] and −25.7 [male-to-male sexual contact and heterosexual contact, females] to −29.0 [heterosexual contact, males]) and then partially rebounded in Q3 (Table 2).

**FIGURE:**
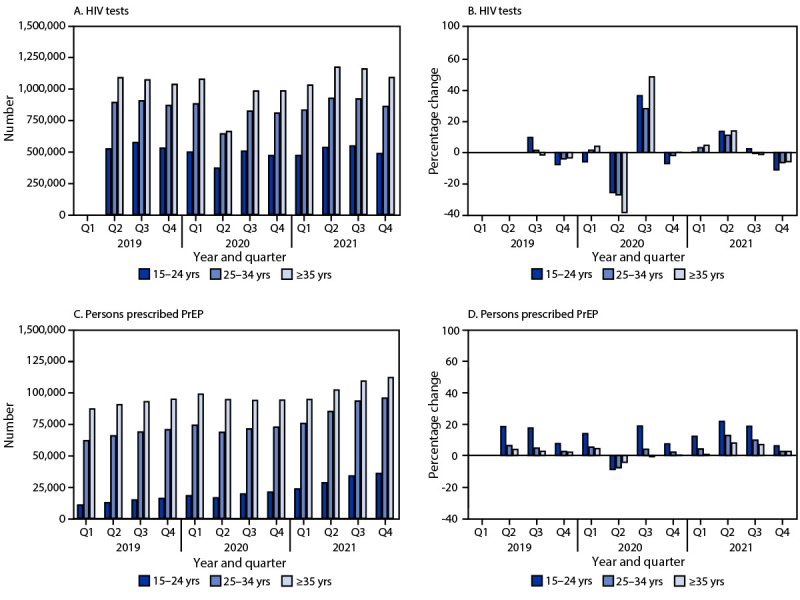
Change in the number of HIV tests (A),* percentage change in the number of HIV tests from quarter to quarter (B),^†^ change in the number of persons prescribed preexposure prophylaxis (C),^§^ and percentage change in the number of persons prescribed preexposure prophylaxis from quarter to quarter (D),^¶^ by age group — United States 2019–2021 **Abbreviations:** PrEP = preexposure prophylaxis; Q1 = quarter 1; Q2 = quarter 2; Q3 = quarter 3; Q4 = quarter 4. * Commercial laboratory HIV antigen/antibody testing data from LabCorp and Quest Diagnostics, 2019–2021. Because data were incomplete for January 2019, the Q1–Q2 change was not calculated. ^†^ The percentage change in the number of HIV tests from Q1 2020 to Q2 2020 was larger among persons aged ≥35 years (−38.6%; 95% CI = −38.8 to −38.4) compared with persons aged 15–24 years (−25.7%; 95% CI = −26.0 to −25.4) and 25–34 years (−27.2%; 95% CI = −27.4 to −27.0). ^§^ IQVIA Real World Data — Longitudinal Prescription Database, 2019–2021. ^¶^ The percentage change in the number of persons prescribed PrEP from Q1 2020 to Q2 2020 was larger among persons aged 15–24 years (−8.9%; 95% CI = −10.8 to −6.9) compared with persons aged 25–34 years (−7.8%; 95% CI = −8.7 to −6.8) and ≥35 years (−4.4%; 95% CI = −5.2 to −3.5).

**TABLE 2:** Number of persons diagnosed with HIV infection, by age, race and ethnicity, and transmission category by quarter* — National HIV Surveillance System, United States, 2019–2020

Characteristic	No. of HIV diagnoses (% change from previous quarter)								2019–2020 EQPC (95% CI)
	2019				2020				
	Q1	Q2	Q3	Q4	Q1	Q2	Q3	Q4	
**Age group, yrs**									
13–24	2,062 (—)	1,964 (−4.8)	1,916 (−2.4)	1,682 (−12.2)	1,702 (1.2)	1,279 (−24.9)	1,570 (22.8)	1,526 (−2.8)	−5.17 (−5.86 to −4.47)
25–34	3,322 (—)	3,398 (2.3)	3,320 (−2.3)	3,018 (−9.1)	3,076 (1.9)	2,314 (−24.8)	2,969 (28.3)	2,894 (−2.5)	−3.13 (−3.66 to −2.59)
≥35	4,104 (—)	4,069 (−0.9)	3,928 (−3.5)	3,692 (−6.0)	3,660 (−0.9)	2,635 (−28.0)	3,366 (27.7)	3,338 (−0.8)	−4.15 (−4.63 to −3.66)
**Race and ethnicity**									
Black or African American	4,036 (—)	3,956 (−2.0)	3,875 (−2.0)	3,584 (−7.5)	3,577 (−0.2)	2,588 (−27.6)	3,353 (29.6)	3,300 (−1.6)	−3.98 (−4.47 to −3.49)
Hispanic or Latino^†^	2,539 (—)	2,524 (−0.6)	2,516 (−0.3)	2,292 (−8.9)	2,276 (−0.7)	1,640 (−27.9)	2,043 (24.6)	2,035 (−0.4)	−4.48 (−5.09 to −3.86)
White	2,385 (—)	2,399 (0.6)	2,246 (−6.4)	2,025 (−9.8)	2,108 (4.1)	1,663 (−21.1)	2,042 (22.8)	2,012 (−1.5)	−3.37 (−4.00 to −2.73)
Other^§^	528 (—)	552 (4.5)	527 (−4.5)	491 (−6.8)	477 (−2.9)	337 (−29.4)	467 (38.6)	411 (−12.0)	−4.50 (−5.82 to −3.16)
**Transmission category** ^¶^									
Heterosexual contact, female	1,424 (—)	1,526 (7.2)	1,425 (−6.6)	1,347 (−5.5)	1,300 (−3.5)	966 (−25.7)	1,182 (22.4)	1,087 (−8.0)	−5.00 (−5.80 to −4.19)
Heterosexual contact, male	720 (—)	682 (−5.3)	653 (−4.3)	614 (−6.0)	600 (−2.3)	426 (−29.0)	511 (20.0)	475 (−7.0)	−6.46 (−7.63 to −5.27)
Male-to-male sexual contact	6,261 (—)	6,146 (−1.8)	6,043 (−1.7)	5,487 (−9.2)	5,614 (2.3)	4,174 (−25.7)	5,399 (29.3)	5,372 (−0.5)	−3.25 (−3.65 to −2.86)
Persons who inject drugs**	1,059 (—)	1,057 (−0.2)	1,020 (−3.5)	920 (−9.8)	896 (−2.6)	645 (−28.0)	789 (22.3)	804 (1.9)	−5.52 (−6.48 to −4.56)

**Abbreviations:** EQPC = estimated quarter percentage change; Q1 = quarter 1; Q2 = quarter 2; Q3 = quarter 3; Q4 = quarter 4.

* Quarters were defined as Q1 (January 1–March 31), Q2 (April 1–June 30), Q3 (July 1–September 30), and Q4 (October 1–December 31).

^†^ Hispanic or Latino persons can be of any race.

^§^ Other includes American Indian or Alaska Native, Asian, Native Hawaiian or other Pacific Islander, and multiracial.

^¶^ Classified based on a hierarchy of the risk factors most likely responsible for HIV transmission; classification is determined based on the person’s sex assigned at birth. Data have been statistically adjusted to account for missing transmission category.

** Includes persons who inject drugs and engage in male-to-male sexual contact.

## Discussion

Compared with the performance of the U.S. HIV prevention and care service system before the COVID-19 pandemic, the system performed as well as it did during the first 2 years of the pandemic when access to services decreased as a result of shutdowns and loss of employer-sponsored health insurance (*1*–*3*). HIV testing and PrEP prescriptions were disrupted during Q2 2020 but rebounded during Q3 after which PrEP prescriptions followed prepandemic trends, increasing each quarter through 2021. The decrease in HIV diagnoses might be attributable to decreases in HIV testing as well as decreases in transmission during the pandemic. Despite the decline in HIV diagnoses, similar proportions of persons receiving a diagnosis were linked to care compared with prepandemic proportions. Although viral load tests decreased in Q2 2020, ART prescriptions remained stable, suggesting that prescriptions were provided without recommended viral load testing.^§§^ This is consistent with guidelines recommending providers and their patients to weigh the risks and benefits of in-person care, including visits for laboratory testing, during periods of high COVID-19 community transmission.^¶¶^

Interventions to increase HIV testing and PrEP use outside of clinical settings were being implemented in the United States before and during the COVID-19 pandemic and can be expanded during future public health emergencies or other periods of decreased health care access. HIV and PrEP self-test kits are in various stages of development, evaluation, and distribution (*8*–*10*). The use of such testing kits, along with health service models that include telehealth clinical services and an expanded role for pharmacies, can provide opportunities for PrEP initiation and continued use over time during periods of decreased access to health care venues.

In 2020, the Coronavirus Aid, Relief, and Economic Security Act appropriated $90 million to Ryan White HIV/AIDS Program (RWHAP) recipients to facilitate response to clients’ COVID-19–related health service needs.*** The Health Resources and Services Administration (HRSA) HIV/AIDS Bureau (HAB) waived certain administrative requirements for RWHAP recipients and subrecipients. These include eligible clients be persons with HIV infection, so that COVID-19 prevention measures could be provided to close contacts who did not have HIV; penalty provisions, including requirements for obligation of funds and core medical services budgets; and the requirement for a nominal charge for clients with incomes above the federal poverty level. Recipients were encouraged to be flexible in client eligibility determinations and recertification processes, including adoption of self-attestation and electronic signatures for jurisdictions that did not already use them. HRSA HAB encouraged adoption of telehealth services and mobile technology to increase access to services.

The findings in this report are subject to at least four limitations. First, although HIV antigen and antibody and viral load tests were not nationally representative, they included more than one half of laboratory tests performed in the United States. Second, IQVIA data were not nationally representative but included prescriptions from 93% of retail pharmacies and 77% of mail-order pharmacies. Third, HIV and viral load testing data were not deduplicated across LabCorp and Quest Diagnostics. A person might have had more than one test result, resulting in an overestimation of persons with an HIV or viral load test result. Finally, viral suppression estimates did not include persons out of care; these persons might have been less likely to be virally suppressed. The viral suppression method in this study differs from the viral suppression measure used to monitor the EHE initiative, which is calculated using NHSS data on all persons with diagnosed HIV infection in the United States. However, viral suppression rates in this study are similar to EHE initiative measures for persons who received care or a viral load test.

The HIV prevention and care service system was resilient during the COVID-19 pandemic. Although HIV testing and PrEP services were disrupted in the spring of 2020, these services started to rebound by summer 2020; ART services for treatment remained unchanged because of interventions such as telehealth and ART home delivery. HIV testing and PrEP provision using self-test kits and nonclinical delivery models are needed to ensure robust prevention services during public health emergencies. Data on the impact of disrupted services and outcomes during the pandemic, along with risk behavior change data, can be used in models to predict the impact on HIV transmission and delays in achieving goals of the EHE initiative. Communities can use this information to assess resources and activities needed to offset decreased prevention services during the pandemic.

**Summary**

**What is already known about this topic?**

HIV service use decreased after the COVID-19 public health emergency declaration in March 2020.

**What is added by this report?**

In 2020, the number of HIV tests and the number of persons prescribed preexposure prophylaxis (PrEP) decreased between the first and second calendar quarters but rebounded by the third quarter. The proportion of persons linked to HIV care, the number prescribed antiretroviral therapy, and the proportion with a suppressed viral load among those tested remained stable during the study period.

**What are the implications for public health practice?**

Innovative service delivery models for HIV testing and PrEP care are needed to ensure that these services are accessible during public health emergencies.

## Footnotes

*
https://www.hiv.gov/federal-response/ending-the-hiv-epidemic/overview

†
https://www.iqvia.com/locations/belgium/library/fact-sheets/real-world-longitudinal-prescription-data

§ The study period for analyses using NHSS data was January 2019–December 2020.

¶ Quarters were defined as Q1 (January 1–March 31), Q2 (April 1–June 30), Q3 (July 1–September 30), and Q4 (October 1–December 31).

** Data included in this study are from 46 jurisdictions (45 states and the District of Columbia) that had complete laboratory reporting for all data years. Linkage to care was defined as having one or more CD4 or viral load tests within 1 month of HIV diagnosis.

†† 45 C.F.R. part 46.102(l)(2); 21 C.F.R. part 56; 42 U.S.C. Sect. 241(d); 5 U.S.C. Sect. 552a; 44 U.S.C. Sect. 3501 et seq.

§§
https://clinicalinfo.hiv.gov/en/guidelines

¶¶
https://clinicalinfo.hiv.gov/sites/default/files/guidelines/documents/guidelines-covid-19-hiv.pdf

***
https://ryanwhite.hrsa.gov/grants/coronavirus
